# Pachydermoperiostosis Presenting With End-Stage Kidney Disease

**DOI:** 10.7759/cureus.62408

**Published:** 2024-06-14

**Authors:** Hunar K Ghai, Sandhya Suresh, Ram Prasad Elumalai

**Affiliations:** 1 General Medicine, Sri Ramachandra Institute of Higher Education and Research, Chennai, IND; 2 Nephrology, Sri Ramachandra Institute of Higher Education and Research, Chennai, IND

**Keywords:** end-stage renal disease (esrd), complementary and alternative medicines, osteoarthropathy, etoricoxib, pachydermoperiostosis

## Abstract

Pachydermoperiostosis, also known as Touraine-Solente-Golé syndrome, is an uncommon hereditary condition. This condition includes skin thickening (pachydermia), abnormalities of the bones (periostosis), and digital clubbing (acropachy). We present a case of complete pachydermoperiostosis who presented with end-stage kidney disease. Chronic tubulointerstitial disease secondary to long-term analgesics and complementary and alternative medications was considered the likely etiology for renal dysfunction. The patient underwent serial hemodialysis followed by arteriovenous fistula surgery. In view of significant synovial inflammation, he was also given a selective COX-2 inhibitor. Pachydermoperiostosis is a rare condition, and although there is no therapy for the condition itself, medicinal or surgical interventions can effectively control its secondary effects.

## Introduction

Pachydermoperiostosis (PDP) is a familial disorder with autosomal dominant inheritance [1]. Typically, it emerges during adolescence, occurring almost exclusively in males, with a male-to-female ratio of 7:1 [2]. Elevated levels of prostaglandin E2 (PGE2) have been suggested as a contributing factor, and treatments are available to suppress this prostaglandin. The disease mainly impacts the bones and joints. Although renal involvement is rare, there have been reports of PDP patients presenting with glomerulonephritis and renal amyloid A amyloidosis [3]. In this context, we report a case of PDP that progressed to end-stage kidney disease.

## Case presentation

**Figure 1:**
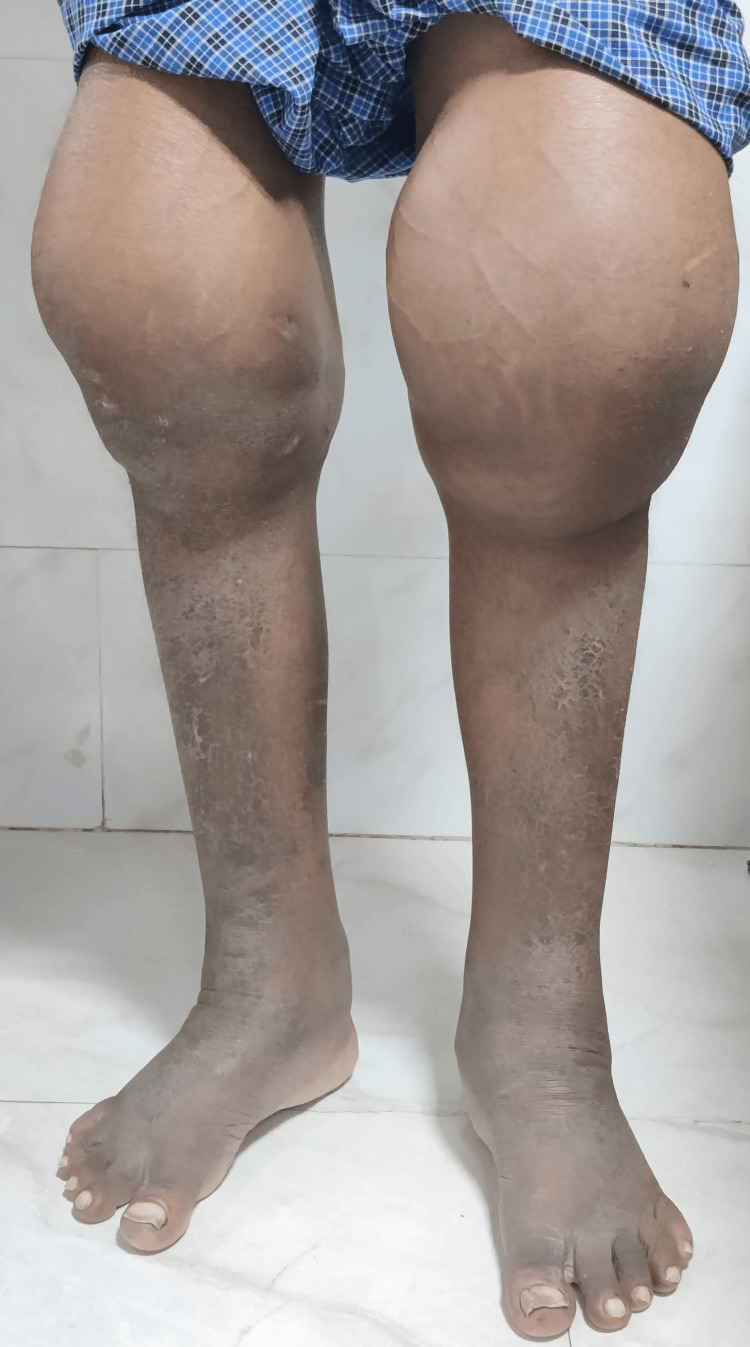
Bilateral knee swelling

**Figure 2:**
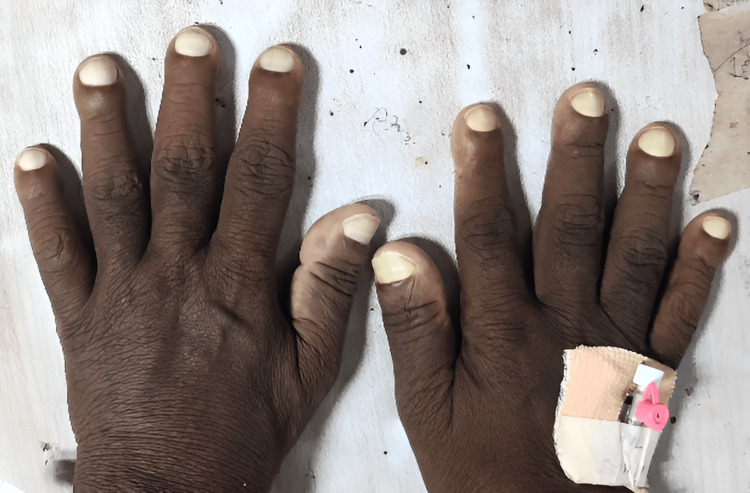
Clubbing in upper limbs

**Figure 3:**
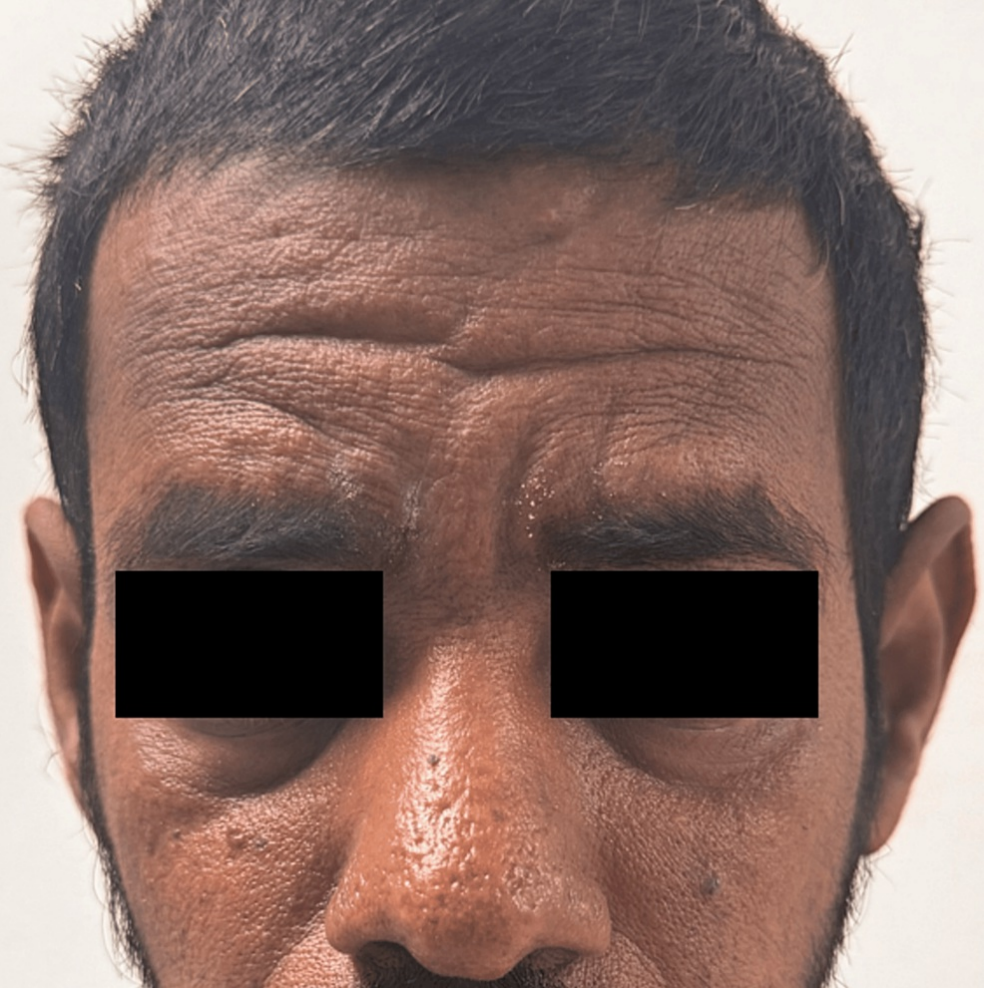
Distinct forehead wrinkles, marked nasolabial folds, and seborrhea

We report the case of a 46-year-old non-smoker and non-alcoholic male with a history of hypothyroidism and hypertension for five years, who presented to our institute with multiple episodes of vomiting associated with nausea and decreased urine output for one week. He had a history of bilateral knee joint pain and swelling for several years for which he was taking long-term analgesics and complementary and alternative medications. On general examination, he had hypertrophic skin changes: thickening of forehead skin with fissuring, periodontal changes, grade 4 clubbing of bilateral fingers and toes, and massive swelling of bilateral knee joints (Figure 1, Figure 2, and Figure 3).

Baseline investigations revealed that hemoglobin (Hb) was 6.1 g/dl, total white blood count (WBC) was 5290 cells/mm^3^, and platelets were 2.26 lakhs/mm^3^. Renal function tests showed blood urea nitrogen 127 mg/dl and creatinine 8.7 mg/dl. The urine routine showed 2+ proteinuria with no active urinary sediment or pyuria. The bone mineral profile revealed phosphorus at 8.1 mg/dl, uric acid at 14.1 mg/dl, calcium at 5.5 mg/dl, albumin at 2.6 mg/dl, and parathyroid hormone levels of 379.10 pg/ml. In view of anemia, further evaluation showed serum iron 76.61 mcg/dl, ferritin 773 ng/ml, and total iron binding capacity 139 mcg/dl, and peripheral smear showed microcytic hypochromic anemia. USG of the abdomen showed a right kidney size of 8.9×3.7 cm and a left kidney size of 8.5×4.4 cm with bilateral increased cortical echoes and poorly maintained corticomedullary differentiation.

In view of uremic symptoms, the patient was initiated on hemodialysis. He was started on activated vitamin D and calcium supplementation for chronic kidney disease (CKD)-mineral bone disease (MBD) with secondary hyperparathyroidism. He was also given Tab Desidustat 100 mg thrice weekly for severe anemia of chronic disease. He underwent arteriovenous fistula surgery and is currently on maintenance hemodialysis. A renal biopsy could not be performed as renal cortical thinning was present with lost corticomedullary differentiation. Possible etiologies for CKD considered included drug-induced chronic tubulointerstitial disease (CTID) and secondary amyloidosis. Given the history of chronic nephrotoxic medication intake and presentation with anemia, MBD, and severe metabolic acidosis with low-grade proteinuria, CTID was considered likely.

The patient had persistent joint swelling and pain which was impairing mobility. Magnetic resonance imaging (MRI) of bilateral knee joints showed gross suprapatellar effusion with hypointense frond-like projections along the synovium. He was advised joint reconstruction surgery. He was started on etoricoxib, a selective COX-2 inhibitor, after which he had a significant reduction in swelling.

## Discussion

PDP, historically known as Touraine-Solente-Golé syndrome or primary hypertrophic osteoarthropathy, is an exceptionally rare medical condition that manifests primarily through digital clubbing, periostosis of tubular bones, and pachydermia. Secondary symptoms may include excessive sweating (hyperhidrosis), joint pain (arthralgia), gastric ulcers, thickened scalp skin (cutis verticis gyrata), drooping eyelids (blepharoptosis), joint fluid buildup, column-like legs, oily skin (seborrhea), and acne [4,5].

The precise etiology of PDP is not completely understood. Nevertheless, the condition has been associated with the chromosomal region 4q33-q34, and mutations in the HPGD gene, which codes for 15-hydroxyprostaglandin dehydrogenase, an enzyme crucial for the breakdown of prostaglandins, have been identified as a contributing factor [4].

Elevated levels of PGE2 due to defective degradation linked to mutations in the HPGD and SLCO2A1 genes are implicated in PDP's development. The degree of pachydermia and associated histological alterations have been correlated with serum PGE2 levels and SLCO2A1 genotypes. PGE2 may replicate the functions of osteoblasts and osteoclasts, potentially resulting in acro-osteolysis and periosteal bone formation. Additionally, prolonged local vasodilation effects of PGE2 might account for digital clubbing [6].

While direct renal involvement in PDP is not a typical clinical feature, there are reports of chronic glomerulonephritis in PDP, with one case progressing to end-stage kidney disease. Another case involved renal complications due to secondary amyloidosis, with chronic inflammation leading to amyloid A protein deposition. In our patient, the kidneys appeared echogenic on ultrasound, rendering renal biopsy unsuitable. Considering the subnephrotic range proteinuria, severe metabolic acidosis, and CKD-MBD in the context of prolonged analgesic and complementary or alternative medicine (CAM) use, drug-induced chronic tubulointerstitial nephritis was suspected as the most likely cause of renal failure.

Drug-induced nephrotoxicity is a significant issue in clinical settings, with drug-related acute kidney injury (AKI) incidence rates reaching up to 60%. The pathophysiology of drug-induced nephrotoxicity involves a multifaceted process often characterized by changes in intraglomerular hemodynamics, reduced tubular secretion, inflammation, uric acid accumulation, rhabdomyolysis, and thrombotic microangiopathy [7].

In many developing countries, traditional medicine is the primary source of healthcare for up to 80% of the population. In industrialized nations, variations of traditional medicine, referred to as CAM, are increasingly utilized for preventive or palliative care. However, CAM can pose significant risks for both acute and chronic renal injuries due to several factors. These include the lack of standardization in the composition and biological activity of nonconventional preparations; the presence of unknown over-the-counter or prescription drugs, hormones, and glandular extracts; contamination of herbal products with pesticides and heavy metals; and the potential for plant identification errors and confusing terminology. The absence of professional oversight leads to underreporting of adverse events and drug interactions, and specific data on systemic and kidney toxicity are limited [7].

Our patient exhibited persistent joint inflammation and was on hemodialysis for end-stage kidney disease. Consequently, etoricoxib, a selective COX-2 inhibitor, was prescribed, as it is considered the treatment of choice for PDP. Increased PGE2 levels, crucial for eicosanoid-mediated inflammatory responses, resulting from defective breakdown pathways, also contribute to PDP pathophysiology [8]. Etoricoxib has demonstrated efficacy in modulating central pain mechanisms and improving pain and function in osteoarthritis. A case report indicated that combining etoricoxib with aescin and synovectomy provided promising results in reducing inflammation and slowing the progression of pachydermia.

## Conclusions

PDP is a rare pathological condition, characterized by a paucity of documented cases detailing its impact on renal function. The prolonged administration of anti-inflammatory agents among afflicted individuals constitutes a contributing factor to the development of chronic renal disease, culminating in end-stage kidney disease. 
